# Trade-Offs and Partitioning Strategy of Carbon Source-Sink During Fruit Development of *Camellia oleifera*

**DOI:** 10.3390/plants14131920

**Published:** 2025-06-23

**Authors:** Yueling Li, Yiqing Xie, Yue Zhang, Xuan Fang, Jian Wang

**Affiliations:** 1 School of Geographical Sciences, Fujian Normal University, Fuzhou 350007, China; 109082022003@student.fjnu.edu.cn (Y.L.); 13385570599@163.com (Y.Z.); 2 Fujian Provincial Key Laboratory for Plant Eco-Physiology, Fujian Normal University, Fuzhou 350007, China; fxuan1992@163.com; 3 Institute of Economic Forestry, Fujian Academy of Forestry, Fuzhou 350012, China; xyqing168@163.com; 4 School of Liberal Arts Education and Art Media, Xiamen Institute of Technology, Xiamen 361021, China

**Keywords:** nonstructural carbohydrates, carbon balance, allometric partitioning theory, fruit development, *Camellia oleifera*

## Abstract

Non-structural carbohydrates (NSCs), the main substrates and energy carriers of plants, play an important role in mediating the source-sink balance of carbon (C). However, the trade-offs in the allocation of NSCs remain unclear at critical stages of fruit development. In this study, we evaluated the dynamic and allometric partitioning characteristics of NSCs at the key stage of fruit development in *Camellia oleifera*. The seed NSCs pool was the highest in the middle stage of rapid fruit expansion, and an inverted “V” shape appeared from July to September and peaked in August. Notably, although the NSC pool of twigs was the smallest and did not change significantly at each stage, the starch pool was the largest. Significant correlations existed between the NSC content of different organs in *C. oleifera* in the early stage of slow development and the middle stage of rapid fruit expansion. In particular, NSC components, both of the twigs in the early stage and of the twigs and seeds in the middle stage, showed significant allometric partitioning relationships. In summary, seeds are the main carbon sink for fruit development trade-offs of *C. oleifera*, and twigs may play an important role in transferring C to seeds at the early and middle stages of fruit development. In the future, attention should be paid to controlling the factors affecting the balance of plant C during the rapid fruit expansion period to ensure high yield.

## 1. Introduction

Non-structural carbohydrates (NSCs) are a key and commonly used indicator for studying the carbon (C) status and allocation dynamics of plants. They are primarily composed of starch and soluble sugars that are inter-convertible and, in conjunction with current photoassimilates, provide energy and C substrates for metabolic processes [1,2,3,4,5]. As the labile fraction and the dominant currency of the C partition, they play essential roles in mediating the C source-sink balance [6,7,8]. Previous studies on NSCs in plants have focused more on the basic physiological functions of NSCs, such as their role in plant photosynthesis, respiration, and growth [1,9,10,11]. Currently, an increasing number of studies are being conducted on the disparity and dynamics of NSCs in different plant species and organs [12,13,14,15,16,17]. Studies have shown significant differences in NSC content among different tree species. This difference may be related to the physiological characteristics of the tree species, adaptability of the growing environment, and demand for C and allocation strategies [6,18]. However, there were significant differences in the content and distribution of NSCs in different plant organs. Generally, leaves, as the main site of photosynthesis, have a relatively high content of NSCs, which is mainly used to support their own metabolic activities and growth [19,20], while other studies have found that trees partition a greater proportion of NSCs to their roots [21]. These results revealed that there were differences not only in NSC content among tree species and NSC allocation among organs but also in NSC components and their changes over time [22]. Prior research has discussed the differences between species, organs, NSC components, and the temporal dynamics of NSCs. However, most studies have almost completely overlooked the effects of plant reproductive organs, such as fruits and seeds, and their growth and development stages on NSCs and their components.

Plant reproduction, one of the most important stages of plant life history, plays a crucial role in ensuring the long-term survival and reproduction of plant populations [23,24,25]. As the key organ for plant reproduction, fruit growth and development regulate the material and energy partitioning of the plant life cycle [26]. It has been found that NSCs were transferred to the fruit in large quantities during fruit ripening [27]. At the same time, as abundant constituents of fruits, the content of NSC components in the fruits differs between species and also with their stage of development [24,28,29]. According to research, starch in fruit starts accumulating in the first 50 days after full bloom and reaches a maximum concentration during the middle phase of fruit development [28]. Recent findings have corroborated that fruits occupy the largest proportion of starch during the fruit growth cessation period in the tree [24]. Considering all of the above, the dynamics and partitioning characteristics of NSCs and their components during fruit development have not been unified in previous studies and are still not well understood. Therefore, tracking the time scale and exploring the knowledge of fruit phenological development is still necessary, as the plant reproduction period is always correlated with lower NSC levels, indicating that there is a trade-off between plant growth and reproduction [30]. In the case of limited resources, the trade-off relationship of plants is usually achieved through allometric partitioning; that is, plants need to make trade-offs between the allometric partitioning of resources to different functional organs or physiological processes [31].

Allometric partitioning is a universal law for the distribution of plant resources and materials in different organs [32]. The allometric exponent is not affected by individual growth and can reflect the changes in resource partitioning patterns of other parts, explaining the growth patterns and adaptive strategies of plants and helping to understand how plants partition their limited resources during growth and reproduction [33,34]. In general, plants allocate more resources to leaves to fix new carbohydrates, the root system to absorb water and nutrients, the stems to provide mechanical support and transport, or the seeds to propagate offspring [35]. Allometric partitioning was originally used to describe the patterns of biomass distribution among different plant organs. It has now been extended to include a wider variety of resource allocation strategy studies [36,37,38,39]. The scaling relationships of N concentration across different plant organs tended to be allometric between leaves and non-leaf organs and isometric between non-leaf organs, while the scaling relationships of P concentration tended to be allometric between roots and non-root organs and isometric between non-root organs [40,41]. The rate of S content variation in roots was faster than that in leaves, branches, and stems, and there was a significant allometric distribution relationship between non-adjacent organs (e.g., leaves and stems) [31]. In previous studies, the inconsistency of allometric partitioning characteristics between different tree species, resources, organs, plant growth and development stages, and phenological periods has been highlighted. Models based on the allometric relationship hypothesis have fewer parameters and better simulation effects, but there is no consistent conclusion regarding this hypothesis in forest ecosystems [31,42,43]. NSCs are essential for the growth and development [41,44] of trees. How to make trade-offs between organ partitioning and component transformation to coordinate tree growth can reflect the functional differences of various organs remains unclear. The study of the allometric partitioning of NSCs at the key stage of plant fruit growth plays a crucial role in the regulation of plant growth and development, resource allocation, and optimization of fruit quality [24,29]. The allometric partitioning characteristics of NSCs involved in the stage of reproduction cannot be simply reflected by scale transformation [2,9]. Further research is needed on the dynamics and allometric partitioning characteristics of NSCs at the key stage of plant fruit growth and development.

*Camellia oleifera* Abel. is an important and unique woody oil-bearing tree species in China with high ecological, economic, and medicinal value [45]. However, most research on *C. oleifera* has focused on resource evaluation, breeding, soil composition, and cultivation techniques [46,47,48], with limited attention paid to its fundamental physiological processes, particularly those governing resource allocation during the economically critical reproductive phase [49,50]. Specifically concerning NSC dynamics, preliminary studies have revealed the temporal and organ-specific patterns of NSCs and their components in the development of *C. oleifera* fruits [49,50,51,52]. The study focused on the fruit development of *C. oleifera* from July to September and showed that both soluble sugar and starch levels in seeds were highest in July; organs with more active metabolism are often allocated more C and thus serve as the main sinks during this stage [51]. However, a different study indicated that the level of starch in *C. oleifera* fruit peaked in September [52], suggesting that the precise dynamics and partitioning characteristics of different organs throughout the fruit development period are still controversial. Crucially, existing studies have either covered only partial developmental stages or have lacked integration with the theoretical framework of allometric partitioning trade-offs [45,46,47,48,49,50,51,52]. The dynamics and allometric partitioning characteristics of NSCs are of great economic significance for increasing yield and optimizing quality and have important ecological significance for understanding the allocation strategy of plant resources. Therefore, further research is still needed during the critical period of growth and development of *C. oleifera* fruit.

Therefore, to bridge this critical knowledge gap, this study investigated the dynamics of NSCs and their components (soluble sugars, starch, and total NSCs) in leaves, twigs, peels, and seeds throughout the entire fruit development period of *C. oleifera*. Particularly, we employed the framework of allometric partitioning theory to analyze the trade-offs in NSCs allocation among these organs at key stages. This integrated approach encompasses the full developmental timeline, multiple organs (including economically vital seeds), and the application of allometric partitioning analysis. We hypothesized that (i) as a woody oil-bearing tree species, *C. oleifera* seeds may be the primary sink during the fruit development period, and NSCs will reach their peak before seed maturation in preparation for heavy carbohydrate consumption in later physiological processes. (ii) There were stage differences in the allometric partitioning characteristics of NSCs and their components, and at the critical stages of fruit development, the allometric partitioning characteristics may be more reflected in an increased investment towards reproductive organs (fruits or seeds) to propagate offspring, thereby ensuring the long-term survival and reproduction of plant populations. In this way, we aim to elucidate the trade-off strategy of C allocation at the organ level during *C. oleifera* fruit development, thereby providing a theoretical basis for improving the cultivation and management of *C. oleifera*.

## 2. Results

### 2.1. The Disparity of NSCs in Camellia oleifera Organs

**Figure 1:**
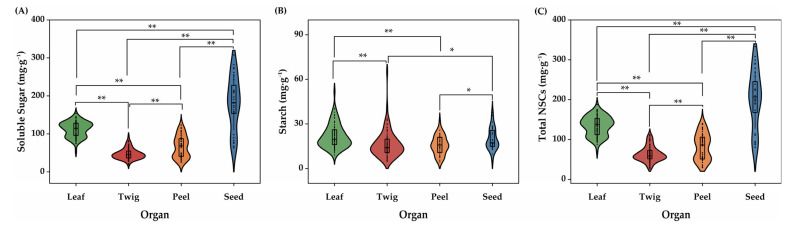
The disparity of NSCs in four *Camellia oleifera* organs. (**A**): Soluble Sugar, (**B**): Starch and (**C**): Total NSCs. **, and * denote the significance level (*p* < 0.01, and 0.05), respectively.

The significant disparity of NSCs components in different *C. oleifera* organs were observed. The changes in soluble sugar content displayed enormously significant differences among the four organs (*p* < 0.01). The seed had the highest soluble sugar content, while the twig had the lowest; in descending order, they were seed > leaf > peel > twig (Figure 1A). Moreover, starch content in leaf and twig, leaf and peel had extremely remarkable differences (*p* < 0.01), while twig and seed, peel and seed had significant differences (*p* < 0.05). The results demonstrated the maximum starch content was in the twig, yet the minimum was in the peel, ranging from maximum to minimum; the sequence is twig > leaf > seed > peel (Figure 1B). Similarly, the variations of total NSCs exhibited a marked variance in the four organs (*p* < 0.01). The range of NSCs content in each organ was 70.35~187.49 mg·g^−1^ in leaves, 26.52~111.81 mg·g^−1^ in twigs, 29.73~139.7 mg·g^−1^ in peels, 80.63~333.14 mg·g^−1^ in seeds. The average content in leaves, twigs, peels, and seeds was 134.22 ± 2.59, 63.39 ± 2.02, 81.63 ± 4.82 and 200.08 ± 12.60 mg·g^−1^, respectively, showing seed > leaf > peel > twig (Figure 1C).

**Figure 2:**
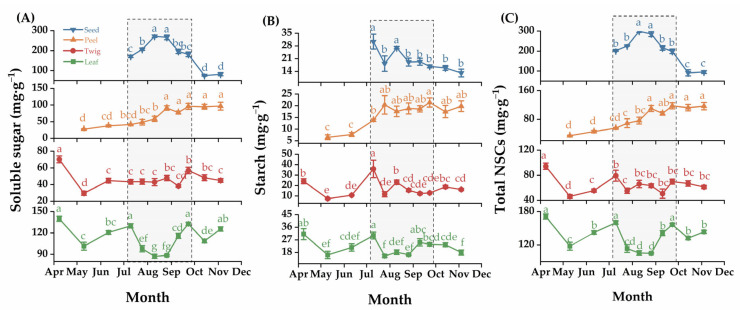
The dynamics of NSC in four *C. oleifera* organs. (**A**): Soluble Sugar, (**B**): Starch and (**C**): Total NSCs. Values are means ± standard errors, and different lowercase letters indicate significant difference (*p* < 0.05).

The contents of soluble sugar, starch, and total NSCs in the four *C. oleifera* organs varied significantly between months. In general, the dynamics of soluble sugar and total NSC content demonstrated an overall similarity throughout the sampling months, while the changes in starch content showed relative complexity from July to September (Figure 2A–C). Specifically, the highest soluble sugar and total NSC concentrations in leaves and twigs were observed in April (*p* < 0.05). The soluble sugar and total NSC content in the peels increased from May to November. The peaks of soluble sugar and total NSCs in seeds were observed in August, significantly higher than in other months (*p* < 0.05), presenting an inverted “V”-shaped change trend (Figure 2A,C). Moreover, the starch content in leaves, twigs, and seeds first decreased and then increased as the months progressed, reaching its highest point in July. The number of peels was constantly increasing. The trends in the other months were similar to those of soluble sugars and total NSCs (Figure 2B).

### 2.2. The Dynamics of NSCs in C. oleifera Organs at the Critical Stage of Fruit Development

**Figure 3:**
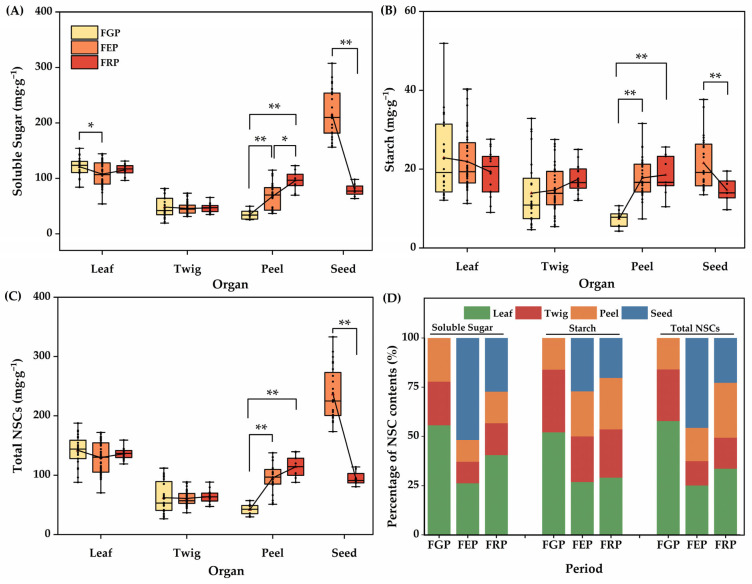
The dynamics of NSC in four *C. oleifera* organs at the critical stage of fruit development. (**A**): Soluble Sugar, (**B**): Starch and (**C**): Total NSCs. (**D**): the percent stacking plot of NSC contents. Abbreviations FGP represents the young fruit growing stage, FEP represents the rapid fruit expanding period, and FRP represents the fruit ripening stage. The lines in the figure connect the average points of the months representing the key stages of fruit development. **, and * denote the significance level (*p* < 0.01, and 0.05), respectively.

Analyzed the critical stage of fruit development, except for the soluble sugar content in the leaf showed a significant decrease from the early stage of slow fruit development to the middle stage of rapid fruit expansion (*p* < 0.05), the changes of other NSC components in leaf and twig exhibited no significant fluctuations during the whole period. Moreover, the soluble sugar, starch, and total NSC contents in the peel showed a constant increase at critical stages of fruit development, while a decrease was observed in seeds from the middle stage of rapid fruit expansion to the late stage of stable fruit maturity. Specifically, the leaf had the minimum soluble content at the middle stage of rapid fruit expansion, which was significantly lower than that at the early stage of slow fruit development (*p* < 0.05), but had no obvious variance between the middle stage of rapid fruit expansion and the late stage of stable fruit maturity (Figure 3A–C).

The percentages of soluble sugar, starch, and total NSCs in the four *C. oleifera* organs were higher in the leaves at the early stage of slow fruit development, all exceeding 50%. Soluble sugar and total NSCs occupied the highest percentage in seeds at the middle stage of rapid fruit expansion, accounting for about 50%, while starch was evenly stored in four organs at the middle stage of rapid fruit expansion and the late stage of stable fruit maturity, at around 25%. The total NSCs were also evenly stored in leaves, peels, and seeds and were higher than those in the twigs at the late stage of stable fruit maturity (Figure 3D).

### 2.3. The Correlation of NSCs in C. oleifera Organs at the Critical Stage of Fruit Development

The correlation between NSCs and their components in the four organs of *C. oleifera* at each fruit development stage was significant.

In the early stage of slow fruit development, there was a positive correlation with the total NSCs in leaves: Ss-Leaf-1, Ss-Twig-1, S-Leaf-1, S-Twig-1, T-Twig-1, Ss-Leaf-2, and a negative correlation with the total NSCs in leaves: Ss-Peel-2, Ss-Seed-2, T-Twig-2, T-Peel-2, and T-Seed-2. There was a positive correlation with the total NSCs in twigs: Ss-Leaf-1, Ss-Twig-1, S-Leaf-1, S-Twig-1, and T-Leaf-1, and a negative correlation with the total NSCs in twigs: Ss-Peel-2, Ss-Seed-2, S-Twig-2, T-Twig-2, T-Peel-2. A significant positive correlation was observed between T-Peel-1 and Ss-Peel-1.

In the middle stage of rapid fruit expansion, there was a significant positive correlation between T-Leaf-2 and S-Leaf-2 and a significant negative correlation between T-Leaf-2 and S-Seed-2. T-Twig-2 was only negatively correlated with Ss-Leaf-1, Ss-Twig-1, S-Leaf-1, T-Leaf-1, T-Twig-1, Ss-Peel-3, T-Peel-3. There was a positive correlation with the total NSCs in peels: Ss-Peel-2, S-Peel-2, and Ss-Leaf-3, and there was a negative correlation with the total NSCs in peels: Ss-Leaf-1, Ss-Twig-1, S-Leaf-1, S-Twig-1, T-Leaf-1, and T-Twig-1. There was a significant positive correlation between T-Sees-2 and Ss-Seed-2 and a negative correlation with the total NSCs in seeds: T-Leaf-1, Ss-Leaf-2, and S-Leaf-2.

**Figure 4:**
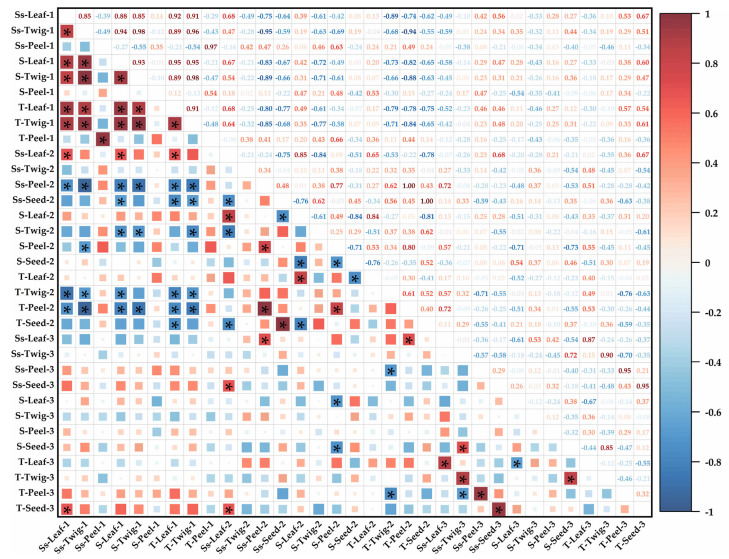
The Pearson’s correlation of each NSC component in the four *C. oleifera* organs. Data were log_10_ transformed before analysis to conform to or near a normal distribution and to improve the normality of the residuals. The color scale of the correlation heat map ranged from −1 to 1. Red indicates a positive correlation (color scale ranges from 0 to 1), while blue represents a negative correlation (color scale ranges from −1 to 0). * denotes significance (*p* < 0.05). 1~3 represent the three stages of *C. oleifera* fruit growth and development processes (1: the early stage of slow fruit development, 2: the middle stage of rapid fruit expansion, 3: the late stage of stable fruit maturity). Abbreviations Ss-Leaf represents the leaf-soluble sugars, Ss-Twig represents the twig-soluble sugars, Ss-Peel represents the peel-soluble sugars, Ss-Seed represents the seed-soluble sugars, S-Leaf represents the leaf-starch, S-Twig represents the twig-starch, S-Peel represents the peel-starch, S-Seed represents the seed-starch, T-Leaf represents the leaf-total NSCs, T-Twig represents the twig-total NSCs, T-Peel represents the peel-total NSCs, and T-Seed represents the seed-total NSCs.

Furthermore, in the late stage of stable fruit maturity, T-Leaf-3 was positively correlated with Ss-Leaf-3 and negatively correlated with S-Leaf-3. T-Twig-3 was positively correlated only with Ss-Twig-3 and S-Seed-3. T-Peel-3 was positively correlated with Ss-Peel-3 and negatively correlated with T-Twig-2 and Ss-Twig-2. Similarly, T-Seed-3 was only positively correlated with Ss-Leaf-1, Ss-Leaf-2, and Ss-Seed-3 (Figure 4).

### 2.4. The Allometric Partitioning Characteristics of NSCs in C. oleifera Organs at the Critical Stage of Fruit Development

**Figure 5:**
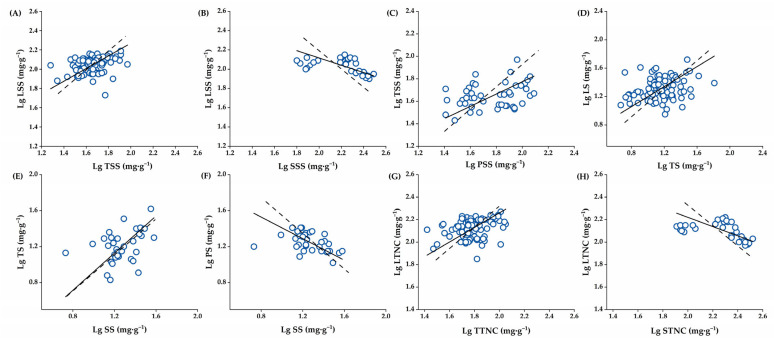
The scatter plot of the allometric allocation relationship of each NSC component in the four *C. oleifera* organs during fruit development. (**A**): The allocation of soluble sugar content between leaves and twigs, (**B**): The allocation of soluble sugar content between leaves and seeds, (**C**): The allocation of soluble sugar content between twigs and peels, (**D**): The allocation of starch content between leaves and twigs, (**E**): The allocation of starch content between twigs and seeds, (**F**): The allocation of starch content between peels and seeds, (**G**): The allocation of total NSCs between leaves and twigs, (**H**): The allocation of total NSCs between leaves and seeds. Abbreviations: LS, Leaf Starch; LSS, Leaf Soluble Sugar; TS, Twig Starch; TSS, Twig Soluble Sugar; PS, Peel Starch; PSS, Peel Soluble Sugar; SS, Seed Starch; SSS, Seed Soluble Sugar; LTNC, Leaf Total Non-structural carbohydrates; TTNC, Twig Total Non-structural carbohydrates; STNC, Seed Total Non-structural carbohydrates. The dashed line indicates a slope of 1.0.

Overall, the absolute values of the scaling exponent (slope, *α*_SMA_) of *α*_Leaf-Twig_ (0.657), *α*_Leaf-Seed_ (−0.379), and *α*_Twig-Peel_ (0.548) for soluble content, *α*_Leaf-Twig_ (0.705) and *α*_Peel-Seed_ (−0.600) for starch content, and *α*_Leaf-Twig_ (0.648) and *α*_Leaf-Seed_ (−0.411) for total NSCs content were all <1.0, while that of *α*_Twig-Seed_ (1.033) for starch content was >1.0. Therefore, the soluble sugar content in twigs and seeds changed faster than that in leaves, and that in peels changed faster than that in twigs. The starch content in twigs changed faster than that in leaves and seeds, and in seeds, it changed faster than that in peels. The NSCs content in twigs and seeds changed faster than that in leaves. In addition, *α*_Twig-Seed_ for starch content was not significantly different from 1.0 and showed isometric relationships, while *α*_Leaf-Twig_, *α*_Leaf-Seed_, and *α*_Twig-Peel_ for soluble sugar content, *α*_Leaf-Twig_ and *α*_Peel-Seed_ for starch content, and *α*_Leaf-Twig_ and *α*_Leaf-Seed_ for total NSCs content showed significant allometric relationships (Table 1, Figure 5A–H).

**Figure 6:**
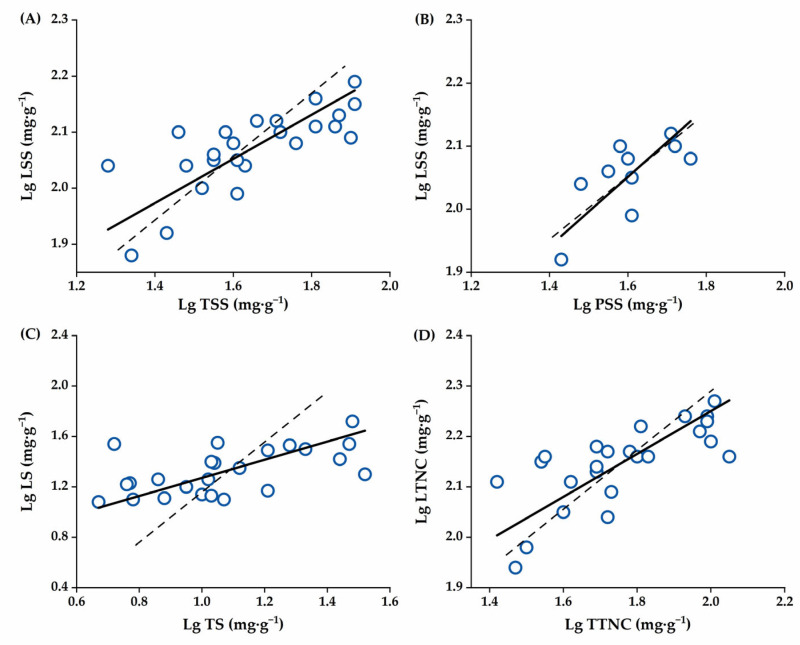
The scatter plot of each NSCs component allocation in the four *C. oleifera* organs at the young fruit growing stage. (**A**): The allocation of soluble sugar content between leaves and twigs, (**B**): The allocation of soluble sugar content between leaves and peels, (**C**): The allocation of starch content between leaves and twigs, and (**D**): The allocation of total NSCs between leaves and twigs. Abbreviations: LS, Leaf Starch; LSS, Leaf Soluble Sugar; TS, Twig Starch; TSS, Twig Soluble Sugar; PSS, Peel Soluble Sugar; LTNC, Leaf Total Non-structural carbohydrates; TTNC, Twig Total Non-structural carbohydrates. The dashed line indicates a slope of 1.0.

Specifically, we divided the development of fruit into three critical stages for analysis. First, the NSCs component allocation characteristics of the four *C. oleifera* organs in the early stage of slow fruit development at 4–6 months are shown in Table 2 and Figure 6A–D. The scaling exponent (slope, α_SMA_) of α_Leaf-Twig_ (0.394) and α_Leaf-Peel_ (0.552) for soluble sugar content, α_Leaf-Twig_ (0.719) for starch content, and α_Leaf-Twig_ (0.425) for total NSCs content were all <1.0. Therefore, the soluble sugar content in twigs and peels changed faster than that in leaves, the starch content in twigs changed faster than that in leaves, and the NSCs content in twigs also changed faster than that in leaves. In addition, α_Leaf-Twig_ and α_Leaf-Peel_ for soluble sugar components and α_Leaf-Twig_ for total NSCs were significantly different from 1.0 and showed allometric relationships, while α_Leaf-Twig_ for starch components was not significantly different from 1.0, which showed an isometric relationship.

**Figure 7:**
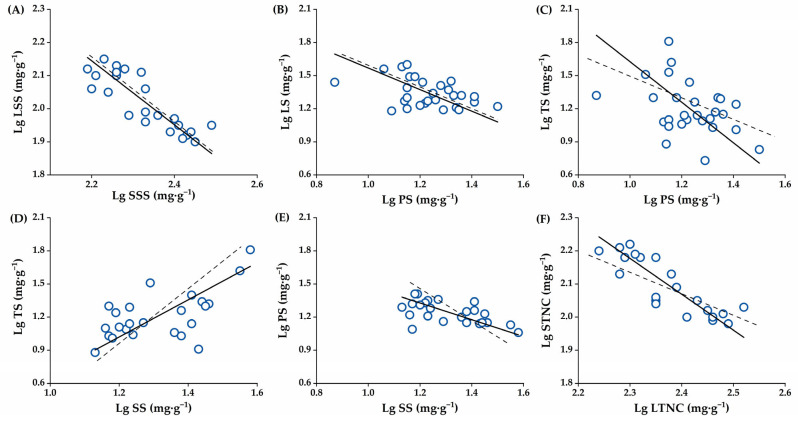
The scatter plot of the allometric allocation of each NSC component in the four *C. oleifera* organs at the rapid fruit expansion stage. (**A**): The allocation of soluble sugar content between leaves and seeds, (**B**): The allocation of starch content between leaves and peels, (**C**): The allocation of starch content between twigs and peels, (**D**): The allocation of starch content between twigs and seeds, (**E**): The allocation of starch content between peels and seeds, (**F**): The allocation of total non-structural carbohydrates (NSCs) between leaves and seeds. Abbreviations: LS, Leaf Starch; LSS, Leaf Soluble Sugar; TS, Twig Starch; TSS, Twig Soluble Sugar; PS, Peel Starch; PSS, Peel Soluble Sugar; SS, Seed Starch; SSS, Seed Soluble Sugar; LTNC, Leaf Total Non-structural carbohydrates; TTNC, Twig Total Non-structural carbohydrates; STNC, Seed Total Non-structural carbohydrates. The dashed line indicates a slope of 1.0.

Second, in the middle stage of rapid fruit expansion at 7–9 months, the absolute value of the scaling exponent (slope, *α*_SMA_) of *α*_Leaf-Seed_ (−0.966) regarding soluble sugar content, *α*_Leaf-Peel_ (−0.977) and *α*_Peel-Seed_ (−0.758) regarding starch content was all <1.0, while that of *α*_Twig-Peel_ (−1.838) and *α*_Twig-Seed_ (1.683) regarding starch content, *α*_Leaf-Seed_ (−1.084) regarding total NSCs content was all >1.0. Therefore, the soluble sugar content in seeds changed more rapidly than that in leaves. The starch content in peels changed faster than in leaves, in seeds changed faster than in peels, and in twigs changed faster than in peels and seeds. For the total NSC content, leaves changed faster than seeds. In addition, α_Leaf-Seed_ regarding soluble sugar content, *α*_Leaf-Peel_ and *α*_Peel-Seed_ regarding starch content, and *α*_Leaf-Seed_ regarding total NSCs content were not significantly different from 1.0 and showed isometric relationships, while *α*_Twig-Peel_ and *α*_Twig-Seed_ regarding starch content showed significant allometric relationships (Table 3, Figure 7A–F).

**Figure 8:**
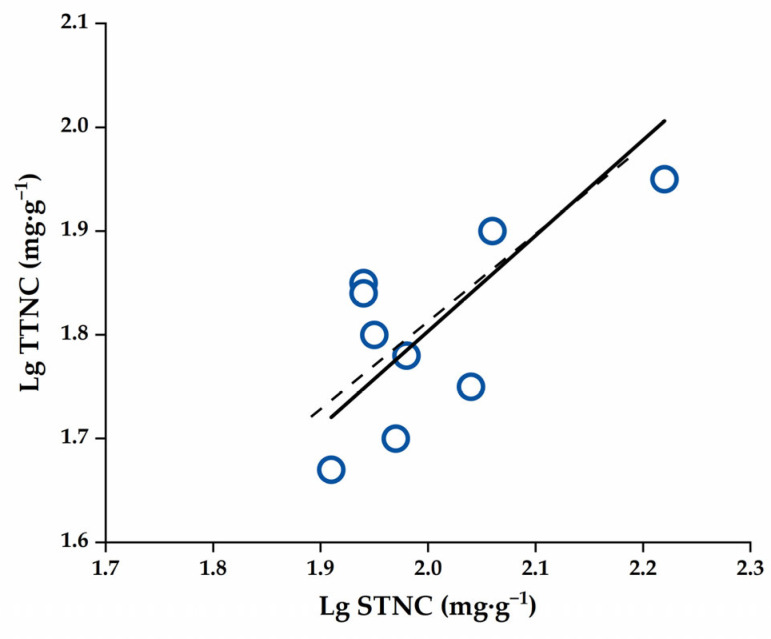
The scatter plot of allometric allocation of each NSC component in four *C. oleifera* organs at the rapid fruit expansion stage. Abbreviations: TTNC, Twig Total Non-structural carbohydrates; STNC, Seed Total Non-structural carbohydrates. The dashed line indicates a slope of 1.0.

Additionally, all key parameters of NSCs component allocation of the four *C. oleifera* organs in the late stage of stable fruit maturity at 10–11 months were described (Table 4), and only twigs and seeds had a remarkable linear positive correlation (*p* < 0.01) and the scaling exponent (slope, *α*_SMA_) of *α*_Twig-Seed_ (0.921) about total NSCs content had no significant difference with 1.0 manifesting an isometric relationship (Table 4, Figure 8).

## 3. Discussion

### 3.1. The Disparity of NSCs in Four C. oleifera Organs

NSCs vary significantly among different organs of plants, and these differences reflect the C partitioning status of the plant, which is a key factor in determining tree growth [42,53]. Most earlier research has shown that leaves tend to have the maximum soluble sugar and total NSC content, while the roots tend to have the highest starch content [10,40,54]. However, there are also different studies in which the total NSCs in branches were higher than in leaves, especially starch fractions [7]. Research on C allocation in plant organs is controversial, and most studies have the limitation of not taking into account reproductive organs. In our study, twigs had the highest starch content, similar to the high starch content of branches in the Amazon forests [7]. But unlike previous findings [10,54], seeds had the highest soluble sugar and NSCs content in our study used as the main C sink, which is consistent with our first hypothesis. Despite this, leaves also maintained a relatively high level of NSCs, although they contained less than seeds. This may be due to the fact that the high content of NSCs in leaves is often interpreted as a buffer pool for C metabolism, and its accumulation is closely related to the C source-pool relationship (supporting the C needs of the reproductive organs) [55,56]. Compared with the seasonal trends of NSC components in the fruit and leaves of pear trees [28] and the dynamic changes of NSC components in the fruiting of *Mangifera indica* [24], the amount of starch in our study was not the largest, but soluble sugars, suggesting that *C. oleifera* regulates growth and reproduction mainly by soluble sugar. The significant differences in the content of NSCs between seeds and leaves, especially the greater difference in soluble sugar content, suggest that seeds are the sink organs and leaves are the source organs, transporting the carbohydrates synthesized from the source organs to the sink organs through the phloem, and converting them into their own substances (such as oil and protein) in the sink for storage [18,57].This indicates that the source-sink relationship in plant C allocation is affected by differences in organ-specific metabolic pathways and the metabolic needs of different organs [58]. During the reproductive phase of the plant, the allocation of NSCs is preferentially tilted towards the reproductive organs to meet their needs for growth and development [24,30]. As key reproductive structures of *C. oleifera*, seeds require a large amount of energy and a C skeleton input during the fruit development stage.

The dynamics of NSCs and their components at the plant organ level are important indicators for measuring the C source and C sink capacity of vegetation, and the quantitative study of the contribution of NSCs dynamics to the C balance and carbon cycle is essential to understanding the survival and growth of plants [21,59,60]. The fluctuation of each NSC component was generally similar in *C. oleifera*, which may indicate the synergistic effect of NSC components in regulating fruit growth and development, which distinguishes the distinct dominant role of each NSC component across different growing months, as in a previous study in a mixed temperate forest in Petersham [22]. This can be explained by the differences in the functional division of labor and metabolic dynamics over time [1]. Unlike the October maximum of twig NSCs for Mediterranean tree species [12], our results showed that twigs reached their peak in April before the growth and development of fruits, followed by a rapid decline when young fruits began to grow, with the same trend in leaves. This phenomenon reflects the sink demand of developing fruits, triggering physiological regulation processes in trees that facilitate the redistribution and metabolism of NSCs from leaves and twigs to support young fruit growth and development [20,24], revealing that fruit growth and development have a non-negligible effect on the dynamics of plant organ NSCs. Meanwhile, seeds and leaves exhibited synchronous but opposite changes from July to September, endorsing previous works that have highlighted that the reproductive period always corresponds to lower NSCs levels [12,30]. Nevertheless, it is worth noting that their study did not directly study plant reproductive organs, but rather the reduction of NSCs levels in other organs. Our research provides direct evidence for this. The observed carbohydrate dynamics during fruit maturation revealed established source-sink relationships in trees, demonstrating the preferential flow of NSCs from leaf source organs to developing fruit sink tissues [61,62]. The inverted “V” pattern of *C. oleifera* seeds revealed a distinctive period of oil transition, showing the species specificity of the growth and reproduction strategies (inter-organ allocation strategies and physiological and ecological functions) [21]. In addition, a unique phenomenon of “holding a baby and conceiving an embryo” (overlapping reproductive phases) in *C. oleifera*, in which flowers and fruits coexisted in a single branch at the beginning of November, was observed. In this study, seed NSCs peaked in August, and it is speculated that NSCs in seeds can not only be utilized to meet the needs of future flower bud expansion and flowering [63], but also to provide feedstock for further conversion into camellia oil. NSCs and their components in peels peaked in September, suggesting that the competitiveness of seeds as predominant sink organs was stronger than that of other functional organs, and NSCs were allocated to the priority supply of growth centers. In summary, seed plants transfer a large amount of photosynthetic products from leaves (sources) to seeds (sinks) during the reproductive growth period to propagate their offspring. When researches considered plant reproductive organs and stages, trade-offs exist between plant growth, storage, and reproduction, with plant reproduction being a powerful sink during this period. Sink organs operate in a priority hierarchy, with seeds receiving the highest NSCs allocation precedence.

### 3.2. The Dynamics of NSCs Within C. oleifera Organs at Critical Stage of Fruit Development

Prior work has focused on the dynamics of seasonal and phenological changes in plant NSCs [28,44,51,52,64], while our study took a stage-specific approach to explore the dynamic changes of organ-level NSCs at the three key stages of fruit development in *C. oleifera*, so as to better explore the C allocation strategy. The results demonstrated that there were no significant changes in the NSC components of *C. oleifera* in the organs, except for the seeds, and the leaves, peels, and seeds were the active organs in the mobilization processes. Moreover, NSCs and their components first accumulate in leaves during the young fruit growing period and would not be completely consumed during the rapid fruit expansion period. The high NSCs and soluble sugar content in the seeds during the growth and development stage of *C. oleifera*, not only reflect the high-investment strategy of *C. oleifera* in the reproductive period but also may reduce seed abscission due to the shortage of stored carbohydrates [65]. However, these findings highlight that it is mainly the source-sink relationship that drives the mobilization of NSCs during the critical period of fruit development [62]. Compared to other studies, the roots and stems of oil peony (Fengdan) still fluctuated during the dormant period, with significant differences in the results of NSCs and soluble sugars [58]. A large amount of NSCs were consumed in the leaves of Phyllostachys heterocycla in July, with nearly 0% remaining [66]. This may be due to the fact that the spatial and temporal heterogeneity of NSC accumulation and depletion patterns within woody plants arises from variations in both the initiation timing and duration of organ-specific metabolic activities [66,67]. In *C. oleifera*, trees accumulated a large amount of NSCs in seeds during the rapid fruit expansion period, accompanied by a temporary decrease in NSCs in other organs to ensure that the development of the seeds is not limited by the C supply. This is in agreement with the prioritization of the C needs of reproductive organs when resources are sufficient and the evolutionary adaptation of plants to reproductive success when resources are limited [59,68].

### 3.3. The NSCs Partitioning Characteristics of Four C. oleifera Organs in Critical Stage with the Allometric Analysis

NSC partitioning plays a critical role in C cycling by shifting the products of photosynthesis among different plant organs, and the differences in NSC partitioning in plants reflect the synergy between organs as well as the plant trade-off strategies [30,69]. Trees do not allocate NSCs to various organs in isolation but rather employ a holistic, inter-organ coordinated partitioning strategy [62]. The objective of this study was to explore the holistic and phased NSCs allometric partitioning characteristics of *C. oleifera* fruit development. Firstly, a correlation analysis of the NSC components in the four organs at the three key stages of *C. oleifera* fruit development was performed. Most of the periods were positively correlated with the early stages of slow fruit development, consistent with the correlation analysis of the growth and development stages of *Xanthoceras sorbifolium* [26]. Compared with the periodic study of NSCs in oil peonies (Fengdan) [58], our study not only revealed that leaves and twigs had a synergistic effect on the NSCs requirement of peels and seeds but also further considered inter-stage correlations, which found coordination between the same organs in the early stage of slow fruit development and the middle stage of rapid fruit expansion. However, no consistent correlation was found between the same and different components of NSCs during the development of *C. oleifera* fruits. Moreover, in contrast to the positive correlation between NSC components in Liu et al.’s study [44], most of the NSC components were negatively correlated with each other between the early stage of slow fruit development and the middle stage of rapid fruit expansion, indicating that there were trade-offs between the transformation of NSCs components in different developmental stages of *C. oleifera* fruit. In the late stage of stable fruit maturity, there was intra-stage specificity. Notably, the negative correlation between leaves and seeds in the early stage of slow fruit development and the middle stage of rapid fruit expansion changed to a positive correlation in the late stage of stable fruit maturity, indicating that there was stage specificity for NSCs partitioning in *C. oleifera* fruit development. In the future, the necessity of studying the stage specificity and organ differences of plant NSCs during fruit development should be considered.

Further studies on the allometric partitioning characteristics of *C. oleifera* fruit development were performed. In previous studies on non-photosynthetic organs and the relationship between photosynthetic and non-photosynthetic organs, the results indicated that the proportion of plant photosynthetic products allocated to non-photosynthetic organs increased under certain conditions [46,70]. Although the reproductive organs of plants have not been explored in previous work, the results are relatively consistent with our findings, and the stems of non-photosynthetic organs of plants play an important role in regulating plant allocation patterns. Overall, the analysis of the fruit development process of *C. oleifera* showed that the allometric partitioning of NSCs and their components was most significant in seeds and twigs. This contradicts research on the isometric relationships between reproductive and vegetative organs [71], but can be explained by the intricate link between plant growth and reproduction [20,24,58]. Specifically, compared with leaves, the allometric partitioning characteristics of twigs and peels were significant during the early stages of slow fruit development. Peels are closely related to fruit maturation, which requires rapid regulation of carbohydrates to meet the needs of specific growth stages, while leaves serve as the principal photosynthetic organs, and partitioning stabilization primarily facilitates the fulfillment of essential metabolic demands and supports fundamental growth processes in plants [24]. Meanwhile, the allometric partitioning characteristic of twigs was also remarkable at the middle stage of rapid fruit expansion, which is inconsistent with the conclusion that fruit as a strong reservoir with high sink strength will attract more NSCs investment [29] but consistent with the findings of studies that high twig NSCs concentrations support high fruit yields [8]. This phenomenon may be attributed to the fact that the concentration of NSCs in twigs is closely related to fruit yield, exhibiting complete C autonomy of fruiting at the level of the whole plant [8,72]. These findings support the notion that NSCs make trade-offs between organ allocation and component transformation to coordinate fruit ripening depending on the needs and physiological state of the fruit at different critical periods of growth and development [28,51]. The presence of stage-specific allometric partitioning of NSCs in *C. oleifera* during fruit development verified our second hypothesis. Interestingly, not only was the investment rate of seeds high, but the allometric partitioning characteristics of twigs were the most significant at the stages of fruit development, indicating the ability of twigs as a key structure to regulate the source-sink relationship that plays an important role in the transfer of photosynthetic products to fruits and seeds. NSCs, as essential energy substances in the process of plant growth and development, are affected by life stages, and we cannot ignore the impacts of the key stage during fruit development on NSCs. In the future, attention should be paid to controlling the internal and external factors that affect the plant C balance during the rapid fruit expansion period.

## 4. Materials and Methods

### 4.1. Study Site Description

**Figure 9:**
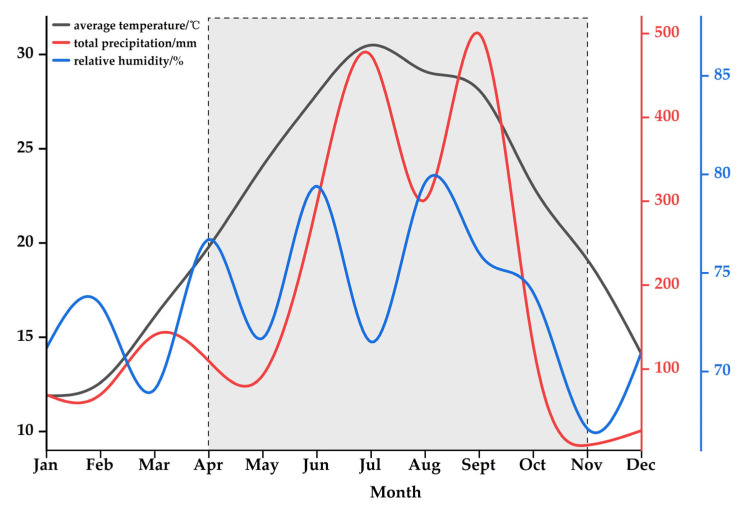
The average temperature, total precipitation, and relative humidity of the study site (Fuzhou, China) in 2023.

This study was conducted at the Tongkou State-owned Forest Farm (E119°14′, N26°09′), Fuzhou, Fujian, at an altitude of 75 m. It is characterized by a typical subtropical monsoon climate with a mean annual temperature (MAT) ranging from 18 to 26 °C. The mean annual precipitation (MAP) is approximately 1300 mm. According to the initial experimental survey in 2022, the basic physicochemical properties of soil, as referred to in Scheffer/Schachtschabel Soil Science [73], were determined as follows: soil pH (METTLER TOLEDO, Shanghai, China) of about 4.79 (acidic), organic carbon (Vario MAX cube, Elementar, Langenselbold, Germany) of 18.98 ± 5.11 mg·g^−1^, total nitrogen of 1.51 ± 0.26 mg·g^−1^, total phosphorus (Vario MAX cube, Elementar, Langenselbold, Germany) of 0.22 ± 0.03 mg·g^−1^, available nitrogen (San Classic, skalar, Delft, The Netherlands) of 125.00 ± 17.20 mg·kg^−1^, and available phosphorus (skalar san++, skalar, Delft, The Netherlands) of 4.22 ± 1.52 mg·kg^−1^ (values are mean ± standard deviation). The plot has not undergone any artificial treatments, such as fertilization or chemical treatments and has been maintained in its natural state. The climate data for 2023 from the China Meteorological Data Service Centre are shown in Figure 9.

### 4.2. Field Sampling

The development processes of *C. oleifera* (Min 43) can be divided into three fundamental stages: the early stage of slow fruit development spans from April to June, the middle stage of rapid fruit expansion lasts from July to September, and the late stage of stable fruit maturity runs from October to November [74,75]. To reveal the mechanisms and strategies of C partitioning, sampling was conducted once per month from April to November 2023, approximately on the 10th day of each month. Additional sampling was conducted on approximately the 25th day of each month from July to September, resulting in two samplings per month during the rapid fruit expansion period. Eight *C. oleifera* trees were studied, and 11 sample collections were conducted, encompassing the entire period of fruit growth, development, and maturation, with a total sampling area of 100 square meters. Multiple twigs, including leaves and fruits, were sampled and preserved using dry ice and subsequently transported to the laboratory. In the laboratory, all harvested samples were wiped clean immediately and categorized into leaves, twigs, peels, and seeds. Notably, data for experimental indicators for peels began to manifest in the initial stages of young fruit growth in May, whereas the seed data were distinct when seeds formed completely and separately in July.

### 4.3. Nonstructural Carbohydrate Analysis

All samples were dried at 60 °C for 48 h (Lab oven, ThermoFisher, Beijing, China) to a constant weight and then ball-milled to a fine powder for analysis (Tissuelyser-24, Shanghai, China). The concentrations of NSC (mg per g dry tissue) were determined using a modified phenol-concentrated sulfuric acid method [76].

We dried pure sucrose (≥99.0%, XILONGS SCIENTIFIC, Shantou, Guangdong, China) at 80 °C until constant weight, weighed 0.1 g samples (accurate to 0.0001 g) for the sucrose standard solution, and plotted the standard curve according to the results of 0, 20, 40, 60, 80, 100, 120, and 140 mg·L^−1^ sucrose standard solution that measured at 490 nm on the spectrophotometer (UV1800, Shimadzu, Kyoto, Japan). (1) Soluble sugars were extracted with 80% ethanol (99.7%, XILONGS SCIENTIFIC, Guangdong, China) for 24 h from approximately 60 mg of dry samples (accurate to 0.0001 g). We separated the mixture at a speed of 4000 revolutions per minute for 10 min to obtain the supernatant, and the remaining residue was re-extracted in 80% ethanol with an additional 5 min of centrifugation. The supernatants were then combined to determine the soluble sugar concentration. (2) To extract the starch, we dried the extracted residue of the soluble sugar at 100 °C for 3 h, then 10 mL of distilled water and 3 mL of 3% hydrochloric acid (99.8%, Sinopharm, Beijing, China) were added to hydrolyze the mixture at 80 °C for 0.5 h in a water bath. The separated supernatant was used to determine the starch content. (3) To measure soluble sugar and starch content, we added 1 mL of the sample solution into test tubes, 1 mL of 20% phenol (≥99.0%, Sinopharm, Beijing, China) solution dissolved in 80% ethanol, and 5 mL of concentrated sulfuric acid (99.8%, Sinopharm, Beijing, China). The mixture was shaken to ascertain the thoroughness of the reaction and allowed to sit for 20 min. After that, we measured the absorbance at 490 nm using a spectrophotometer. We expressed both soluble sugars and starch on the same scale (i.e., glucose equivalents) and summed them to obtain the total NSCs. The calculation formulas for soluble sugars and starch are as follows:

$$\mathrm{Soluble}\mathrm{sugar}\;(mg/g)=\frac{C\times V\times D}{W\times 1000\;}$$

*C* represents the sugar concentration (mg/L) measured using a standard curve.

*V* represents the total volume (mL) of the sample extract;

*D* is the dilution factor;

*W* represents the dry weight (g) of each sample.

The formula for calculating starch content was the same.

### 4.4. Statistical Analysis

One-way analysis of variance (ANOVA) and Duncan’s multiple-range tests were used to analyze the significant differences in NSCs and their components in the four organs. Pearson’s correlation analysis was used to determine the correlations between different NSCs components in the four organs of *C. oleifera*. The allometric index and constants were calculated using the method of standardized spindle regression analysis (standardized major axis estimation, SMA) in SMATR (Standardized Major Axis Tests and Routines) ver. 2.0 software (Evolution & Ecology Research Centre, School of Life and Environmental Sciences, The University of Sydney, Sydney, Australia), together with the confidence interval of the traits regression slope, and the slopes were then tested for heterogeneity. Data were log_10_ transformed before analysis to conform to or near a normal distribution and improve the normality of the residuals. This approach allows for the evaluation of the behavior of one variable in relation to another testing the hypotheses about these relationships and measuring how they vary between samples. Diagrams were drawn using OriginPro 2024b (OriginLab Corporation, Northampton, MA, USA).

## 5. Conclusions

In summary, our results provide a profile of the dynamics and partitioning characteristics of NSC components during the critical period of the whole *C. oleifera* fruit development. Concerning the partitioning trade-offs for NSCs, seeds were the main soluble sugar, and total NSCs sink within the largest investment in the whole fruit development process. The inverted “V”-shaped change from July to September revealed a distinctive period of oil transition. An abundance of NSCs was obtained in the middle stage of rapid fruit expansion, which provided a key substrate for glycolipid transformation in the late stage of stable fruit maturation. The association between early and middle fruit development was significant, and the allometric partitioning characteristics in seeds were significant during the entire fruit development period, providing sufficient energy and material reserves to support reproduction. Additionally, in the allocation patterns of various stages of fruit development, twigs, as the main starch reservoir of *C. oleifera* fruit development, fluctuated little in months and stages, but the phased and holistic allometric partitioning characteristics were remarkable and played a vital role in the adjustment of the internal source-sink structure of fruit development. Our research improves the understanding of *C. oleifera* growth strategies and NSCs partitioning characteristics, which may help enhance fruit quality, assist in simulating C balance, and provide actionable insights for optimizing cultivation and harvest. In the future, attention should be paid to the factors that affect plant growth in cultivation management (such as nutrient management) during pre-peak and peak sink demand and precision harvesting for oil yield, controlling the internal and external factors affecting the balance of plant C during the rapid fruit expansion period to ensure yield.

## Figures and Tables

**Table 1:** Key parameters of NSC components allocation in four plant organs of *Camellia oleifera* by standard major axis (SMA) regression.

Index	Organ (Y–X)	N	R^2^	*p*	*α*_SMA_	95% CI	*β*_SMA_	95% CI	*P*_1.0_
Soluble Sugar (mg·g^−1^)	**Ss_Leaf-Twig_**	**87**	**0.089**	**	**0.657**	**(0.534**, **0.806)**	**0.955**	**(0.731**, **1.180)**	**
	Ss_Leaf-Peel_	47	0.005	ns	−0.433	(−0.582, −0.322)	2.807	(2.574, 3.040)	**
	**Ss_Leaf-Seed_**	**33**	**0.229**	**	**−0.379**	**(−0.520**, **−0.276)**	**2.874**	**(2.603**, **3.146)**	**
	**Ss_Twig-Peel_**	**47**	**0.116**	*	**0.548**	**(0.415**, **0.725)**	**0.678**	**(0.401**, **0.956)**	**
	Ss_Twig-Seed_	33	0.034	ns	−0.445	(−0.633, −0.313)	2.629	(2.272, 2.987)	**
	Ss_Peel-Seed_	33	0.087	ns	−0.788	(−1.111, −0.559)	3.608	(2.993, 4.223)	ns
Starch (mg·g^−1^)	**S_Leaf-Twig_**	**87**	**0.056**	*	**0.705**	**(0.572**, **0.867)**	**0.495**	**(0.319**, **0.670)**	**
	S_Leaf-Peel_	47	0.012	ns	−0.685	(−0.920, −0.511)	2.113	(1.870, 2.355)	*
	S_Leaf-Seed_	33	0.052	ns	0.717	(0.506, 1.017)	0.405	(0.076, 0.734)	ns
	S_Twig-Peel_	47	0.063	ns	1.091	(0.819, 1.453)	−0.105	(−0.480, 0.270)	ns
	**S_Twig-Seed_**	**33**	**0.132**	*	**1.033**	**(0.739**, **1.444)**	**−0.113**	**(−0.566**, **0.341)**	**ns**
	**S_Peel-Seed_**	**33**	**0.170**	*	**−0.600**	**(−0.833**, **−0.432)**	**2.009**	**(1.752**, **2.266)**	**
Total NSCs (mg·g^−1^)	**T_Leaf-Twig_**	**87**	**0.086**	**	**0.648**	**(0.528**, **0.795)**	**0.965**	**(0.726**, **1.204)**	**
	T_Leaf-Peel_	47	0.02	ns	−0.441	(−0.591, −0.329)	2.942	(2.695, 3.190)	**
	**T_Leaf-Seed_**	**33**	**0.184**	*	**−0.411**	**(−0.568**, **−0.297)**	**3.041**	**(2.732**, **3.351)**	**
	T_Twig-Peel_	47	0.044	ns	0.571	(0.428, 0.763)	0.704	(0.387, 1.020)	**
	T_Twig-Seed_	33	0.021	ns	−0.462	(−0.659, −0.324)	2.829	(2.448, 3.211)	**
	T_Peel-Seed_	33	0.098	ns	−0.749	(−1.054, −0.533)	3.658	(3.064, 4.251)	ns

Notes: N, sample number; R^2^, the coefficient of determination; ns denotes no significant difference; ** and * denote the significance level (*p* < 0.01 and 0.05), respectively. *α*_SMA_, slope (i.e., scaling exponent). *β*_SMA_, intercept. CI, confidence interval. ** and * in *P*_1.0_ denote significant differences between the slope of the equation and 1.0 at *p* < 0.01 and 0.05, respectively; ns denotes no significant difference. The bold term indicates that the *p*-value is significant (*p* < 0.05).

**Table 2:** Key parameters of NSC components allocation in four plant organs of *C. oleifera* of young fruit growing stage by standard major axis (SMA) regression.

Month	Index	Organ (Y–X)	N	R^2^	*p*	*α*_SMA_	95% CI	*β*_SMA_	95% CI	*P*_1.0_
4–6	Soluble Sugar (mg·g^−1^)	**Ss_Leaf-Twig_**	**24**	**0.567**	**	**0.394**	**(0.296**, **0.525)**	**1.421**	**(1.231**, **1.611)**	**
		**Ss_Leaf-Peel_**	**10**	**0.473**	*	**0.552**	**(0.315**, **0.969)**	**1.169**	**(0.643**, **1.694)**	*
		Ss_Twig-Peel_	10	0.074	ns	1.060	(0.516, 2.179)	−0.018	(−1.294, 1.258)	ns
	Starch (mg·g^−1^)	**S_Leaf-Twig_**	**24**	**0.311**	**	**0.719**	**(0.502**, **1.029)**	**0.553**	**(0.261**, **0.844)**	**ns**
		S_Leaf-Peel_	10	0.009	ns	1.127	(0.537, 2.366)	0.212	(−0.688, 1.111)	ns
		S_Twig-Peel_	10	0.345	ns	1.184	(0.637, 2.201)	−0.041	(−0.714, 0.631)	ns
	Total NSCs(mg·g^−1^)	**T_Leaf-Twig_**	**24**	**0.547**	**	**0.425**	**(0.317**, **0.570)**	**1.400**	**(1.177**, **1.623)**	**
		T_Leaf-Peel_	10	0.302	ns	0.608	(0.322, 1.150)	1.098	(0.394, 1.802)	ns
		T_Twig-Peel_	10	0.133	ns	1.121	(0.556, 2.259)	−0.115	(−1.492, 1.263)	ns

Notes: N, sample number; R^2^, the coefficient of determination; ns denotes no significant difference; ** and * denote the significance level (*p* < 0.01 and 0.05), respectively. *α*_SMA_, slope (i.e., scaling exponent). *β*_SMA_, intercept. CI, confidence interval. ** and * in *P*_1.0_ denote significant differences between the slope of the equation and 1.0 at *p* < 0.01 and 0.05, respectively; ns denotes no significant difference. The bold term indicates that the *p*-value is significant (*p* < 0.05).

**Table 3:** Key parameters of NSC components allocation in four plant organs of *C. oleifera* of rapid fruit expanding stage by standard major axis (SMA) regression.

Month	Index	Organ (Y–X)	N	R^2^	*p*	*α*_SMA_	95% CI	*β*_SMA_	95% CI	*P*_1.0_
7–9	Soluble Sugar (mg·g^−1^)	Ss_Leaf-Twig_	47	0.005	ns	0.864	(0.643, 1.161)	0.589	(0.159, 1.019)	ns
		Ss_Leaf-Peel_	28	0.022	ns	−0.632	(−0.932, −0.428)	3.162	(2.706, 3.618)	*
		**Ss_Leaf-Seed_**	**24**	**0.720**	**	**−0.966**	**(−1.218**, **−0.766)**	**4.269**	**(3.743**, **4.794)**	**ns**
		Ss_Twig-Peel_	28	0.065	ns	0.700	(0.478, 1.024)	0.404	(−0.091, 0.898)	ns
		Ss_Twig-Seed_	24	0.098	ns	−1.087	(−1.636. −0.722)	4.165	(3.102, 5.227)	ns
		Ss_Peel-Seed_	24	0.042	ns	1.838	(1.207, 2.798)	−2.449	(−4.300, −0.598)	**
	Starch (mg·g^−1^)	S_Leaf-Twig_	47	0.005	ns	0.637	(0.474, 0.855)	0.571	(0.340, 0.802)	**
		**S_Leaf-Peel_**	**28**	**0.172**	*	**−0.977**	**(−1.399**, **−0.682)**	**2.545**	**(2.101**, **2.990)**	**ns**
		S_Leaf-Seed_	24	0.090	ns	0.854	(0.567, 1.287)	0.206	(−0.271, 0.683)	ns
		**S_Twig-Peel_**	**28**	**0.155**	*	**−1.838**	**(−2.642**, **−1.279)**	**3.465**	**(2.619**, **4.311)**	**
		**S_Twig-Seed_**	**24**	**0.370**	**	**1.683**	**(1.193**, **2.375)**	**−1.000**	**(−1.780**, **−0.220)**	**
		**S_Peel-Seed_**	**24**	**0.341**	**	**−0.758**	**(−1.078**, **−0.534)**	**2.239**	**(1.879**, **2.598)**	**ns**
	Total NSCs (mg·g^−1^)	T_Leaf-Twig_	47	0.003	ns	0.896	(0.667, 1.205)	0.495	(0.011, 0.978)	ns
		T_Leaf-Peel_	28	0.067	ns	−0.695	(−1.016, −0.475)	3.433	(2.914, 3.951)	ns
		**T_Leaf-Seed_**	**24**	**0.742**	**	**−1.084**	**(−1.355**, **−0.868)**	**4.671**	**(4.095**, **5.248)**	**ns**
		T_Twig-Peel_	28	0.000	ns	0.795	(0.537, 1.178)	0.288	(−0.326, 0.903)	ns
		T_Twig-Seed_	24	0.068	ns	−1.259	(−1.906, −0.831)	4.770	(3.497, 6.043)	ns
		T_Peel-Seed_	24	0.015	ns	1.780	(1.163, 2.724)	−2.286	(−4.136, −0.436)	**

Notes: N, sample number; R^2^, the coefficient of determination; ns denotes no significant difference; ** and * denote the significance level (*p* < 0.01 and 0.05), respectively. *α*_SMA_, slope (i.e., scaling exponent). *β*_SMA_, intercept. CI, confidence interval. ** and * in *P*_1.0_ denote significant differences between the slope of the equation and 1.0 at *p* < 0.01 and 0.05, respectively; ns denotes no significant difference. The bold term indicates that the *p*-value is significant (*p* < 0.05).

**Table 4:** Key parameters of NSC components allocation in four plant organs of *C. oleifera* of fruit ripening period by standard major axis (SMA) regression.

Month	Index	Organ (Y–X)	N	R^2^	*p*	*α*_SMA_	95% CI	*β*_SMA_	95% CI	*P*_1.0_
10–11	Soluble Sugar (mg·g^−1^)	Ss_Leaf-Twig_	16	0.073	ns	0.590	(0.348, 0.999)	1.086	(0.544, 1.628)	*
		Ss_Leaf-Peel_	9	0.052	ns	0.449	(0.204, 0.985)	1.175	(0.403, 1.946)	*
		Ss_Leaf-Seed_	9	0.010	ns	0.338	(0.152, 0.753)	1.409	(0.829, 1.990)	**
		Ss_Twig-Peel_	9	0.246	ns	1.080	(0.529, 2.205)	−0.461	(−2.116, 1.195)	ns
		Ss_Twig-Seed_	9	0.243	ns	0.814	(0.398, 1.663)	0.103	(−1.117, 1.324)	ns
		Ss_Peel-Seed_	9	0.095	ns	0.754	(0.348, 1.630)	0.522	(−0.714, 1.759)	ns
	Starch (mg·g^−1^)	S_Leaf-Twig_	16	0.059	ns	1.628	(0.958, 2.768)	−0.744	(−1.864, 0.377)	ns
		S_Leaf-Peel_	9	0.081	ns	−1.159	(−2.520, −0.533)	2.730	(1.479, 3.982)	ns
		S_Leaf-Seed_	9	0.000	ns	−0.703	(−1.571, −0.315)	2.087	(1.347, 2.826)	ns
		S_Twig-Peel_	9	0.006	ns	0.877	(0.393, 1.957)	0.124	(−0.862, 1.110)	ns
		S_Twig-Seed_	9	0.138	ns	0.532	(0.249, 1.133)	0.611	(0.094, 1.128)	ns
		S_Peel-Seed_	9	0.108	ns	−0.607	(−1.306, −0.282)	1.949	(1.349, 2.548)	ns
	Total NSCs (mg·g^−1^)	T_Leaf-Twig_	16	0.116	ns	0.433	(0.258, 0.725)	1.355	(0.934, 1.775)	**
		T_Leaf-Peel_	9	0.206	ns	0.337	(0.163, 0.700)	1.438	(0.886, 1.990)	**
		T_Leaf-Seed_	9	0.215	ns	0.252	(0.122, 0.522)	1.626	(1.225, 2.026)	**
		T_Twig-Peel_	9	0.122	ns	1.230	(0.574, 2.635)	−0.721	(−2.838, 1.395)	ns
		**T_Twig-Seed_**	**9**	**0.451**	*	**0.921**	**(0.494**, **1.714)**	**−0.038**	**(−1.259**, **1.184)**	**ns**
		T_Peel-Seed_	9	0.081	ns	0.748	(0.344, 1.626)	0.556	(−0.728, 1.840)	ns

Notes: N, sample number; R^2^, the coefficient of determination; ns denotes no significant difference; ** and * denote the significance level (*p* < 0.01 and 0.05), respectively. *α*_SMA_, slope (i.e., scaling exponent). *β*_SMA_, intercept. CI, confidence interval. ** and * in *P*_1.0_ denote significant differences between the slope of the equation and 1.0 at *p* < 0.01 and 0.05, respectively; ns denotes no significant difference. The bold term indicates that the *p*-value is significant (*p* < 0.05).

## Data Availability

The original contributions presented in this study are included in the article. Further inquiries can be directed to the corresponding author.

## Abbreviations

The following abbreviations are used in this manuscript:

NSCs	Non-structural carbohydrates
C	Carbon
